# Keyhole Mini-Craniotomy Middle Fossa Approach for Tegmen Repair: A Case Series and Technical Instruction

**DOI:** 10.1055/a-2514-7338

**Published:** 2025-02-10

**Authors:** Syed M. Adil, Tanner J. Zachem, Jordan K. Hatfield, Jihad Abdelgadir, Kimberly Hoang, Patrick J. Codd

**Affiliations:** 1 Department of Neurosurgery, Duke University Medical Center, Durham, North Carolina, United States; 2 Department of Neurosurgery, Emory University School of Medicine, Atlanta, Georgia, United States

**Keywords:** cerebrospinal fluid leak, keyhole craniotomy, middle cranial fossa approach, mini-craniotomy, minimally invasive, otorrhea, tegmen defect

## Abstract

**Background and Importance** Tegmen defects associated with cerebrospinal fluid (CSF) leaks are a rare pathology that can result in severe complications if left untreated. There is no universal optimal surgical algorithm for repair, although the most common techniques are the middle fossa craniotomy (traditionally 25 cm ^2^ in area), the transmastoid approach, or both. Here, we describe successful use of a keyhole mini-craniotomy, only 6 cm ^2^ in area, without mastoidectomy or days of lumbar drainage.

**Clinical Presentation** Three patients presented with right-sided CSF otorrhea and hearing loss, with varying sizes of tegmen defects and associated encephaloceles. Keyhole craniotomies measuring 3 × 2 cm were used to perform a multilayer repair comprising an intradural collagen dural substitute, extradural fascial graft, extradural collagen dural substitute, fibrin sealant, and sometimes bony reconstruction using partial thickness craniotomy grafting. All patients were discharged on postoperative day 1 or 2, with no recurrence of symptoms at 6 months.

**Conclusion** The keyhole craniotomy approach does not sacrifice the extent of operative access for this pathology. This minimally invasive approach can likely be used more often without need for concomitant mastoidectomy, ultimately enabling shorter hospital stays and more rapid recovery.

## Background and Importance

Tegmen defects with cerebrospinal fluid (CSF) leakage may be spontaneous or secondary to trauma, surgery, congenital abnormalities, tumors, idiopathic intracranial hypertension, or chronic ear disease and infection. 1 2 3 4 5 6 Presentation includes conductive hearing loss, otitis media, imbalance, tinnitus, CSF otorrhea, and chronic headaches. 1 4 7 8 For CSF leaks, early operative treatment is important in avoiding serious complications (hearing loss, seizures, meningitis, neurological deficits, etc.). 7 9 The algorithm for repair of tegmen defects remains open for debate, 9 although four approaches comprise the cornerstones of treatment 4 : middle cranial fossa, transmastoid, a combination of these two, or middle ear obliteration. Here, we present the use of keyhole mini-craniotomies alone to successfully treat three patients.

## Clinical Presentation: Case Series

### Clinical Presentations

**Fig. 1:**
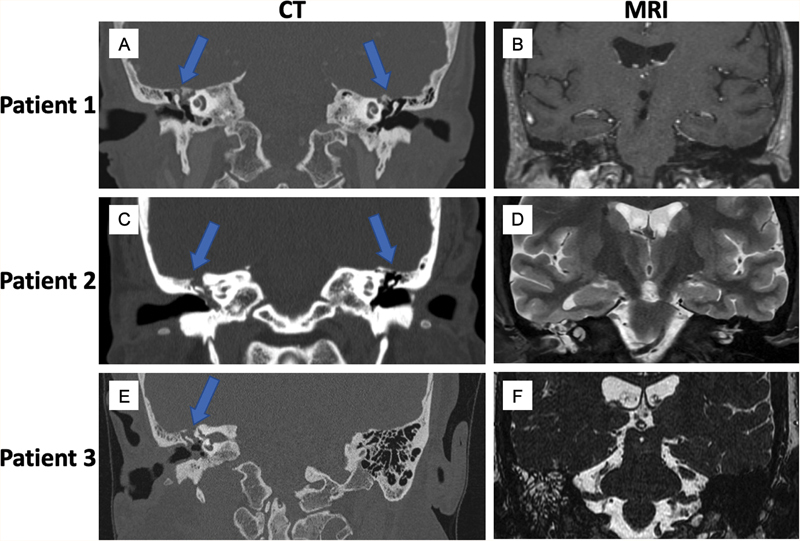
(A, C, E) Preoperative coronal computed tomographies (CTs) demonstrating multiple tegmen defects bilaterally. (B, D, F) Preoperative magnetic resonance imaging (MRI) demonstrating suspected right-sided encephaloceles through tegmen defects.

The three cases were similar in their etiology, presentation, and imaging. All three were female patients, aged 57 to 69 years old, presenting with right-sided hearing loss and auricular fullness. They were evaluated by otologists, who noted frequency-dependent mild to severe hearing loss on the right side. They were diagnosed with chronic otitis media with effusions and thus underwent placement of tympanostomy tubes. After this, all three women had months of persistent CSF drainage from the right ear, reproducible on exam. Thin-cut computed tomography (CT) and magnetic resonance imaging (MRI) scans showed right-sided tegmen dehiscence (largest ∼10 mm), encephaloceles along the middle cranial fossa, and mastoid effusions (representative images in Fig. 1 , with two also having tegmen thinning or small defects on the left without encephaloceles). Patients consented to the publication of their image(s). Per institutional requirements, no institutional review board review was required for this operation. Patients provided informed consent to undergo this procedure with the attending neurosurgeon.

### Operative Technique

**Fig. 2:**
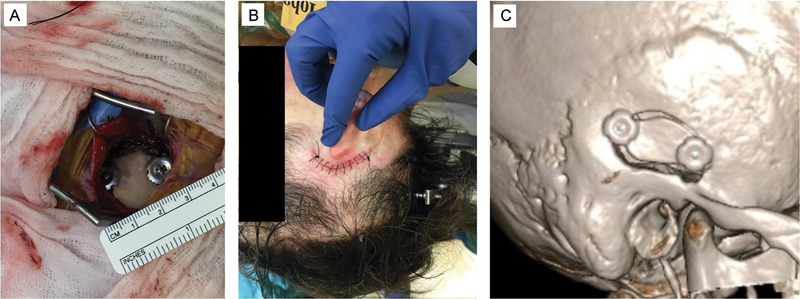
Representative views of intraoperative craniotomy (A), incision at closure (B), and postoperative three-dimensional computed tomography scan showing the right-sided craniotomy (C).

In our first case, we placed a lumbar drain and did not use any hyperosmolar agents, whereas in the latter two cases, we did not place a drain, instead using mannitol and slight reduction of end-tidal CO _2_ . Patients were positioned supine, in a Mayfield clamp, with the middle fossa floor perpendicular to the ground. Navigation based on MRI and CT was utilized to plan an approximately 3-cm curvilinear incision above the ear ( Fig. 2B ), for a planned 3 × 2 cm craniotomy centered along the petrous ridge. During exposure, we harvested a 2 × 2 cm ^2^ temporalis fascia graft before visualizing temporal bone. After making one small burr hole with an acorn bit, we turned the mini-craniotomy with a B1 footplate (see Fig. 2A for craniotomy in vivo and Fig. 2C for 3D-postoperative CT reconstruction). A 3-mm diamond burr was used to drill down the lateral edge of the bone and expose the middle fossa floor. We performed extradural dissection without use of retractors, gently working along the petrous ridge under microscopic visualization posteriorly-to-anteriorly to protect the greater superficial petrosal nerve (GSPN). Facial nerve monitoring showed no changes.

**Fig. 3:**
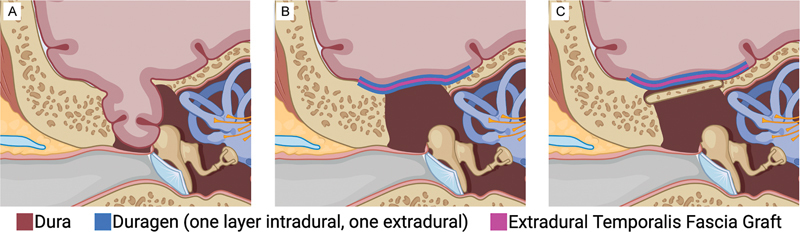
Illustration of multilayered tegmen repair with both intradural (DuraGen inlay) and extradural (temporalis fascia graft, DuraGen onlay, fibrin sealant not pictured, and bone graft) components. (A) pre-repair, (B) multilayered tegmen repair, (C) multilayered tegmen repair with split-thickness bone graft placed. Created with BioRender.com.

**Fig. 4:**
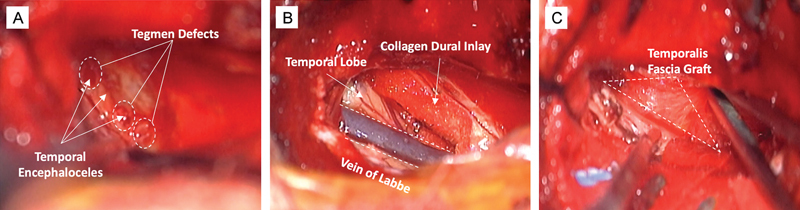
(A) Three tegmen defects identified extradurally, prior to reduction of encephaloceles. (B) Visualization of vein of Labbe via keyhole craniotomy and dural inlay after completion of intradural portion, prior to watertight closure. (C) Fascial graft placed extradurally.

In the first case, the dural defect with encephalocele was just medial to the arcuate eminence and superior semicircular canal, and in the latter two cases, the defects were lateral. CSF was seen copiously pouring out of the defects. The encephaloceles were resected and reduced. For intradural repair, a 2-cm dural opening was created along the lateral temporal dura and a subtemporal approach was then carried out to inspect the middle fossa floor dura. A collagen dural substitute (DuraGen, Integra LifeSciences Corp) inlay graft was placed to cover all dural defects, extending past the bony defects. Then, the temporalis fascia graft was placed in an extradural onlay fashion, again extending past the bone defects. Watertight closure was achieved of the iatrogenic dural opening using 6–0 prolene suture. A third layer using another DuraGen onlay was then placed. The whole repair area was then coated with a fibrin tissue sealant, with care to avoid transit into the middle or inner ear structures conceptualized in Fig. 3 . With microscopic view shown in Fig. 4 . In one case with a larger bony defect, a split thickness bone graft from the craniotomy was also fashioned and placed over this reconstruction (conceptualized in Fig. 3 ). Closure was done in anatomical layers.

### Postoperative Course

The first patient was maintained with 15 to 20 mL/h of CSF drainage for 24 hours, whereas the latter two were not. All patients were discharged on postoperative day 1 or 2. A 2-week levetiracetam taper and 4-day dexamethasone taper were prescribed. At 6 months postoperatively, all three patients remained free of otorrhea.

## Discussion

### The Keyhole Technique

We accomplish the goal of surgery with a 3 × 2 cm (6 cm ^2^ ) craniotomy. The traditional middle fossa craniotomy (MFC) is approximately 5 × 5 cm, 10 and even in more modern case series, they are around 4 × 4 cm 4 11 or 4.5 × 4.5 cm, 12 13 leading to craniotomy surface areas of 16 to 25 cm ^2^ —approximately three to four times larger than ours. Additionally, it is common to use larger incisions, especially when combining with a transmastoid approach (e.g., 6 cm 10 11 ). Some groups use a longer pterional incision. 12 13

Neuronavigation allows for targeting the keyhole approach to pathology specific to a given patient. This is especially valuable when defects are small and numerous, as in some of our cases. Despite the small incision, a generous temporalis fascia graft may still be harvested while minimizing temporalis disruption. Ultimately, the keyhole craniotomy does not limit visualization of critical middle cranial fossa structures (petrous ridge, GSPN, arcuate, etc.). Equally important, the keyhole does not preclude a thorough intradural exploration and repair of defects. When indicated for larger defects (generally >1 cm 14 ), it is also still possible to employ rigid reconstruction of the skull base through this small opening. Even with keyhole repairs, we learned that a lumbar drain is not usually necessary (as others have demonstrated, 13 although practice patterns still vary), further reducing admission duration, discomfort, and cost.

The keyhole concept for this pathology is gaining popularity. Hernandez-Montero et al have employed MFCs of 3 × 2 cm, although they most commonly use a combined transmastoid approach and do not advocate for the MFC alone in their algorithm. 15 Walia et al use a 4 × 2 cm craniotomy, although always part of a combined approach. 10 In their review, Tolisano and Kutz recommend a craniotomy size of approximately 3 × 3 cm. 16 Roehm et al use an expanded burr hole with an endoscopic approach and titanium reconstruction, although keep a lumbar drain in postoperatively, with discharge typically on postoperative day 4 to 5. 17

### Choosing a Surgical Approach: When Not to Use the Keyhole

The transmastoid approach is useful for posterior fossa bony defects, large encephaloceles extending into the mastoid, and small (<1 cm) defects in frail patients who may not tolerate even minimal temporal lobe manipulation. It will still be limited to more posterior/lateral pathology and may put the ossicular chain at risk. 17 Middle ear obliterations should be reserved for patients with already poor hearing and no alternative options. 4 The locations of bony defects in the middle fossa should not preclude a keyhole craniotomy; rather, they may determine its specific location.

## Conclusion

We describe the use of a keyhole mini-craniotomy for tegmen defect repair, without need for mastoidectomy, days of lumbar drainage, or extended hospital stay. This gives excellent cosmesis, reduced tissue disruption, and minimal temporal lobe retraction in carefully selected patients.

## References

[1] Kenning T J Willcox T O Artz G J Schiffmacher P Farrell C J Evans J J Surgical management of temporal meningoencephaloceles, cerebrospinal fluid leaks, and intracranial hypertension: treatment paradigm and outcomes Neurosurg Focus 2012 32 06 E6 10.3171/2012.4.FOCUS1265 22655695

[2] Wahba H Ibrhaim S Youssef T A Management of iatrogenic tegmen plate defects: our clinical experience and surgical technique Eur Arch Otorhinolaryngol 2013 270 09 2427 2431 23179929 10.1007/s00405-012-2260-8

[3] Braca J A III Marzo S Prabhu V C Cerebrospinal fluid leakage from tegmen tympani defects repaired via the middle cranial fossa approach J Neurol Surg B Skull Base 2013 74 02 103 107 24436896 10.1055/s-0033-1333616 PMC3699214

[4] Marchioni D Bonali M Alicandri-Ciufelli M Rubini A Pavesi G Presutti L Combined approach for tegmen defects repair in patients with cerebrospinal fluid otorrhea or herniations: our experience J Neurol Surg B Skull Base 2014 75 04 279 287 25093152 10.1055/s-0034-1371524 PMC4108494

[5] Markou K Goudakos J Franco-Vidal V Vergnolles V Vignes J R Darrouzet V Spontaneous osteodural defects of the temporal bone: diagnosis and management of 12 cases Am J Otolaryngol 2011 32 02 135 140 20392531 10.1016/j.amjoto.2009.12.003

[6] Brodie H A Thompson T C Management of complications from 820 temporal bone fractures Am J Otol 1997 18 02 188 197 9093676

[7] Rao A K Merenda D M Wetmore S J Diagnosis and management of spontaneous cerebrospinal fluid otorrhea Otol Neurotol 2005 26 06 1171 1175 16272936 10.1097/01.mao.0000179526.17285.cc

[8] Merchant S N McKenna M J Neurotologic manifestations and treatment of multiple spontaneous tegmental defects Am J Otol 2000 21 02 234 239 10733190 10.1016/s0196-0709(00)80015-0

[9] Carlson M L Copeland W R III Driscoll C L Temporal bone encephalocele and cerebrospinal fluid fistula repair utilizing the middle cranial fossa or combined mastoid-middle cranial fossa approach J Neurosurg 2013 119 05 1314 1322 23889140 10.3171/2013.6.JNS13322

[10] Walia A Lander D Durakovic N Shew M Wick C C Herzog J Outcomes after mini-craniotomy middle fossa approach combined with mastoidectomy for lateral skull base defects Am J Otolaryngol 2021 42 01 102794 33130529 10.1016/j.amjoto.2020.102794 PMC8048087

[11] McNulty B Schutt C A Bojrab D Babu S Middle cranial fossa encephalocele and cerebrospinal fluid leakage: etiology, approach, outcomes J Neurol Surg B Skull Base 2020 81 03 268 274 32500001 10.1055/s-0039-1688793 PMC7253310

[12] Alwani M Bandali E Van Buren L Yates C W Nelson R F Audiologic improvement following MCF approach for spontaneous cerebrospinal fluid leaks Otol Neurotol 2019 40 08 1026 1033 31157725 10.1097/MAO.0000000000002302

[13] Nelson R F Roche J P Gantz B J Hansen M R Middle cranial fossa (MCF) approach without the use of lumbar drain for the management of spontaneous cerebral spinal fluid (CSF) leaks Otol Neurotol 2016 37 10 1625 1629 27631830 10.1097/MAO.0000000000001208

[14] Porto E Sun H Revuelta-Barbero J M Surgical management of spontaneous middle cranial fossa defects: a systematic review and meta-analysis of available reconstructive techniques and materials Neurosurg Rev 2023 46 01 41 36703023 10.1007/s10143-023-01947-z

[15] Hernandez-Montero E Caballero E García-Ibanez L Surgical management of middle cranial fossa bone defects: meningoencephalic herniation and cerebrospinal fluid leaks Am J Otolaryngol 2020 41 04 102560 32505907 10.1016/j.amjoto.2020.102560

[16] Tolisano A M Kutz J W Jr Middle fossa approach for spontaneous cerebrospinal fluid fistula and encephaloceles Curr Opin Otolaryngol Head Neck Surg 2019 27 05 356 360 31335556 10.1097/MOO.0000000000000560

[17] Roehm P C Tint D Chan N Brewster R Sukul V Erkmen K Endoscope-assisted repair of CSF otorrhea and temporal lobe encephaloceles via keyhole craniotomy J Neurosurg 2018 128 06 1880 1884 28799867 10.3171/2017.1.JNS161947

